# Xnrs and Activin Regulate Distinct Genes during *Xenopus* Development: Activin Regulates Cell Division

**DOI:** 10.1371/journal.pone.0000213

**Published:** 2007-02-14

**Authors:** Joana M. Ramis, Clara Collart, James C. Smith

**Affiliations:** Wellcome Trust/Cancer Research UK Gurdon Institute and Department of Zoology, University of Cambridge, Cambridge, United Kindgom; Baylor College of Medicine, United States of America

## Abstract

**Background:**

The mesoderm of the amphibian embryo is formed through an inductive interaction in which vegetal cells of the blastula-staged embryo act on overlying equatorial cells. Candidate mesoderm-inducing factors include members of the transforming growth factor type β family such as Vg1, activin B, the nodal-related proteins and derrière.

**Methodology and Principle Findings:**

Microarray analysis reveals different functions for activin B and the nodal-related proteins during early *Xenopus* development. Inhibition of nodal-related protein function causes the down-regulation of regionally expressed genes such as *chordin*, *dickkopf* and *XSox17*α/β, while genes that are mis-regulated in the absence of activin B tend to be more widely expressed and, interestingly, include several that are involved in cell cycle regulation. Consistent with the latter observation, cells of the involuting dorsal axial mesoderm, which normally undergo cell cycle arrest, continue to proliferate when the function of activin B is inhibited.

**Conclusions/Significance:**

These observations reveal distinct functions for these two classes of the TGF-β family during early *Xenopus* development, and in doing so identify a new role for activin B during gastrulation.

## Introduction

The mesoderm of the amphibian embryo arises through an inductive interaction in which cells of the vegetal hemisphere act on overlying equatorial cells [1]. Of the several mesoderm-inducing factors that have been discovered, most are members of the transforming growth factor type β family. These include activin [2]–[4], Vg1 [5], [6], five nodal-related proteins [7]–[9], and derrière [10]. Although these factors have similar abilities to induce gene expression in isolated animal pole regions, they are differently expressed in the embryo (see above references) and under some experimental conditions have different abilities to exert long-range effects [11], [12]. In addition, each exerts different effects at different concentrations [7], [13]. The challenge now is to elucidate the individual roles of these proteins within the embryo and to ask how their actions are coordinated.

Some attempts along these lines have been made, and it proves that although each of the factors is essential for normal development, their roles differ. For example, ablation of the maternal transcripts encoding Vg1 causes a reduction in anterior and dorsal development and the down-regulation of genes such as *chordin, cerberus* and *noggin* [6]. Of the zygotically-expressed inducing factors, depletion of activin also causes axial defects [3], [14], [15], although these are less severe than those caused by loss of Vg1, and inhibition of derrière activity causes just posterior defects [10]. Simultaneous inhibition of the activities of all the nodal related proteins, by expression of Cerberus-short, causes loss of mesoderm [16], [17] and the down regulation of genes such as *Chordin* and *Pintallavis* [18]. The requirements of the individual nodal related proteins have not been studied in detail, although injection of antisense morpholino oligonucleotides directed against Xnr1 causes defects in left-right axis determination [19].

Here we perform microarray analyses of gene expression in embryos in which activin or nodal-related signalling has been inhibited. We find that activin and the nodal-related proteins regulate distinct and almost completely non-overlapping sets of genes, with those regulated by the nodal-related genes tending to be expressed in a more restricted pattern than those regulated by activin. It further proved that the nodal-related proteins often regulate the expression of genes involved in regional specification, while activin particularly regulates genes involved in the control of the cell cycle. Consistent with this observation, we find that inhibition of activin B in the early embryo causes dorsal axial mesodermal cells to fail to exit the cell cycle: the results of others [20]–[22] suggest that it is the continued proliferation of these cells that underlies the gastrulation defects observed in such embryos.

## Results

### Microarray results

In an effort to understand the different requirements for activin B and the nodal-related genes during *Xenopus* development, we have carried out microarray analyses of gene expression in embryos in which signalling by the two classes of factor has been disrupted. Activin signalling was blocked using an antisense morpholino oligonucleotide [3], and nodal-related signalling by Cerberus-short, a truncated form of Cerberus [17]. Our microarray slides comprise 10,898 probes designed to recognise sequences derived from a large scale *Xenopus tropicalis* EST project [23]. These arrays also recognise *X. laevis* transcripts [24].

**Figure 1:**
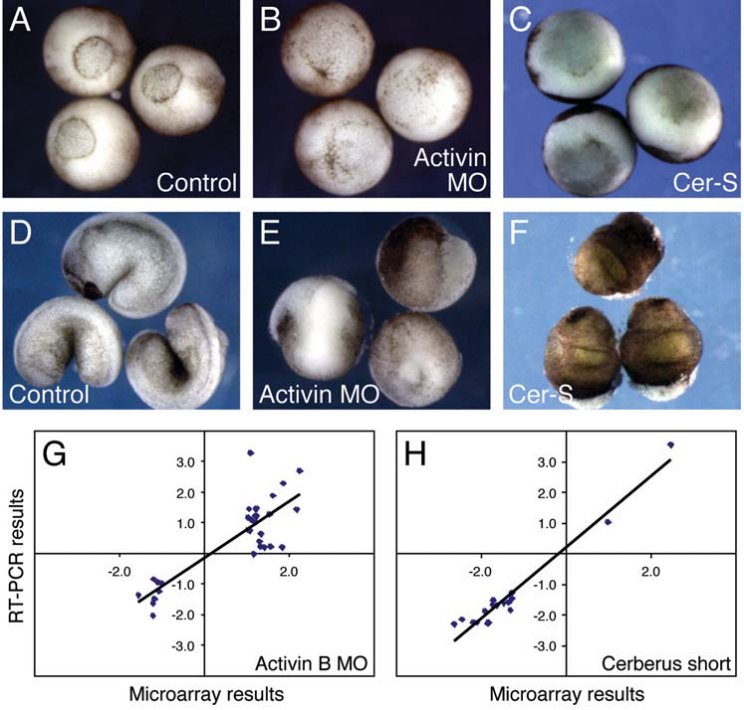
Inhibition of activin B and nodal-related protein function causes distinct phenotypes and results in the differential regulation of different classes of gene. (A,D) Control embryos (here injected with water; those injected with mMO1 look identical) at stage 11 (A) and 26 (D). (B,E) Embryos injected with MO3, and which therefore lack activin B activity. (B) Stage 11; (E) stage 21. Note the delay in gastrulation and the failure to form a proper axis. (C,F) Embryos injected with Cerberus-short RNA, and which therefore lack nodal-related activity. Note the failure to involute and the formation of a radially symmetrical structure. (G,H). Correlation between microarray and PCR results.

For each series of experiments *Xenopus laevis* embryos from three different spawnings were injected with RNA encoding Cerberus-short (150 pg into each blastomere at the four-cell stage) or with antisense morpholino oligonucleotide MO3 (50 ng into the one-cell stage) (samples), or with water or antisense morpholino oligonucleotide mMO1 (50 ng) (controls). These doses of Cerberus-short RNA and MO3 were based on previous work [3], [16] and were chosen so as to yield a strong phenotype in which gastrulation was substantially or completely inhibited. In an effort to identify early and perhaps direct targets of activin and the nodal-related proteins, embryos were cultured to stage 10.5 for RNA isolation and some were allowed to develop to later stages to confirm that embryos displayed the expected phenotypes (Fig. 1A–F). Each microarray slide was hybridised with a 1∶1 mixture of sample and control cDNAs, each labelled with a different dye. Each hybridisation was repeated with the Cy3 and Cy5 dyes ‘swapped’, so that six microarray slides were hybridised for each experiment.

Transcripts recognised by the oligonucleotides were considered to be differentially expressed when (i) they showed at least a two fold difference (sample *versus* control) in expression levels in at least four out of the six microarrays and (ii) were significantly different (q = 0; see Experimental procedures). In embryos in which activin B signalling was inhibited, 40 oligonucleotides fulfilled these rigorous criteria, of which 8 were down regulated, and in those in which nodal signalling was inhibited, 20 oligonucleotides (representing 18 genes) were differentially expressed, of which 17 were down regulated (Table 1). The up regulation of *Cerberus* in the latter experiment is probably due to the introduction of Cerberus-short mRNA into these embryos. Only *Sizzled*, which encodes an inhibitor of the Tolloid Proteinase [25], was differentially expressed in both types of embryo.

**Table 1:** Genes regulated by activin B and nodal-related proteins in the *Xenopus* embryo.

Qiagen Xt oligo name	Gene name	Accession No. *X. tropicalis*	Accession No. *X. laevis*	Log_2_ (sample/control) microarray	Log_2_ (MO3/mMO1)RT-PCR	Log_2_ (CerS/control)RT-PCR	Expression pattern at stage 10.5
**Genes regulated by activin B**							
Xt_10009473	PBK	CR761713	BC088936	2.3	2.7	−0.1	Ubiquitous (this paper)
Xt_10009228	RPN2	CR848133	BC046727	2.2	1.4	−0.1	Ubiquitous (this paper)
Xt_10000757	GADD45G	CR761710	BC078567	1.9	2.3	0.2	Ubiquitous (this paper)
Xt_10004273	TPX2	CX840441	AF244546	1.8	0.2	−0.2	Ubiquitous (this paper)
Xt_10000182	MAPKBP1	AL853880; AL901553	BC076779	1.6	1.9	−0.3	ND
Xt_10006424	unknown	CT030473	no homology	1.6	ND	ND	Ubiquitous (this paper)
Xt_10005146	H2BFS	CR760086	XLHISH3A	1.6	0.2	0.2	Ubiquitous (this paper)
Xt_10009545	ADIPOQ	DT438351; DT438350	BC094476	1.5	1.3	−0.1	ND
Xt_10006410	unknown	DR834759; DR834758	no homology	1.5	ND	ND	ND
Xt_10000971	Serpina3	BC087988	BC084845	1.5	ND	ND	ND
Xt_10000346	H1FOO	CR761180	X13855	1.4	0.2	0.4	Ubiquitous (this paper)
Xt_10004307	unknown	AL790455; BX693495	no homology	1.4	ND	ND	ND
Xt_10010739	BC052883	CX363787; CX363786	AF035443	1.3	0.6	0.0	Ubiquitous (this paper)
Xt_10008973	DNMT1	CT025477	BC072774	1.3	0.2	0.3	Ubiquitous (see Fig. 2)
Xt_10005756	GPR4	CR761039	AY766161	1.3	0.4	−0.5	Ubiquitous (this paper)
Xt_10000636	unknown	AL956096; BX753658	BC085023	1.3	ND	ND	ND
Xt_10005344	RASD1	DR842169; DR842168	BC081268	1.2	1.2	−0.1	ND
Xt_10000635	PCOLN3	BX708936	BC068657	1.2	1.5	−0.1	Ubiquitous (this paper)
Xt_10005362	Sizzled	AL639345	AF136184	1.2	1.4	1.0	Restricted [25]
Xt_10001337	nucleoplasmin	NM_001016938	BC072778	1.2	1.2	−0.1	Ubiquitous (this paper)
Xt_10008633	KRT24	CX745012; CX745011	BC043901	1.2	1.0	0.1	Ubiquitous (this paper)
Xt_10003301	unknown	DR871833; DR871832	no homology	1.2	ND	ND	ND
Xt_10008086	PCQAP	CX911575; CX911574	BC070536	1.2	0.0	0.2	ND
Xt_10007252	unknown	CX961230; CX961229	BC054976	1.2	ND	ND	Ubiquitous (this paper)
Xt_10004134	TUBA1	CT030272	Z31591	1.1	1.1	−0.1	Ubiquitous (this paper)
Xt_10002761	unknown	DR880099; DR880098	no homology	1.1	ND	ND	Ubiquitous (this paper)
Xt_10000615	unknown	CR761187	BC097911	1.1	3.3	−0.2	Ubiquitous (this paper)
Xt_10008957	FAM3A	CR761057	BC108550	1.1	0.7	0.1	Ubiquitous (this paper)
Xt_10004044	Eomesodermin	CX814795; CX814794	BC084243	1.1	1.4	−0.7	Restricted [41]
Xt_10002067	C2orf28	CF591510	BC094117	1.0	0.8	−0.1	Ubiquitous (this paper)
Xt_10008956	Cdc6	CR761778	AY222352	1.0	1.1	0.1	Ubiquitous (this paper)
Xt_10004730	unknown	BQ390504; BQ390503	no homology	1.0	ND	ND	Ubiquitous (this paper)
Xt_10002154	MPDU1	CR761821	BC108439	−1.5	−1.4	0.0	Ubiquitous (this paper)
Xt_10009727	DHCR7	BQ394956; BQ394955	BC054203	−1.2	−1.7	−0.5	Ubiquitous (this paper)
Xt_10000076	cyclin D1	BQ522031; BQ522030	BC041525	−1.2	−2.0	0.3	Ubiquitous (this paper)
Xt_10002938	MRPL12	CR855493	BC084828	−1.2	−0.9	−0.2	Ubiquitous (this paper)
Xt_10010347	EMP2	CT025318	BC106297	−1.2	−1.5	0.1	Ubiquitous (this paper)
Xt_10009006	SOX2	CR760314	AF005476	−1.1	−1.0	0.3	Ubiquitous [42]
Xt_10008667	ATP1A1	CR926442	U49238	−1.1	−1.3	−0.6	Restricted [42]
Xt_10005487	FKBP1B	CT025367	AB006678	−1.0	−1.0	0.2	Ubiquitous (this paper)
**Genes regulated by nodal-related proteins**							
Xt_10003376	unknown	DN089489; DN089488	no homology	3.2	ND	ND	ND
Xt_10002006	Cerberus	NM_203515	BC081277	2.5	−0.4	3.6	Restricted [43]
Xt_10005362	Sizzled	CR761702	AF059570	1.0	1.4	1.0	Restricted [25]
Xt_10010306	unknown	DN030301; DN030300	no homology	−3.0	ND	ND	ND
Xt_10008637	darmin	CX493718	BC055979	−2.8	ND	ND	Restricted [44]
Xt_10000401	HEX	CR761571	U94837	−2.6	0.1	−2.3	Restricted [45]
Xt_10005916	GATA4	NM_001016949	DQ096869	−2.4	−0.2	−2.2	Restricted [42]
Xt_10001409	Xsox17-beta	BX762953	BC070615	−2.2	0.0	−2.3	Restricted [46]
Xt_10000180	Xsox17-beta	CR848411	BC070615	−1.8	0.0	−2.3	Restricted [46]
Xt_10009950	Xdkk-1	NM_001016283	AF030434	−2.1	0.3	−2.3	Restricted [47]
Xt_10009377	GATA6	CT030595	BC082349	−1.9	−0.8	−1.9	Restricted [48]
Xt_10009394	Xsox17-alpha	BC074527	BC106403	−1.8	0.1	−2.3	Restricted [46]
Xt_10000572	Xiro3	BC067972	AF027175	−1.7	−1.4	−1.7	Restricted [49]
Xt_10010791	Xiro3	BC067972	AF027175	−1.6	−1.4	−1.7	Restricted [49]
Xt_10000293	Frzb precursor	CR761513	U78598	−1.7	−0.4	−1.5	Restricted [42]
Xt_10006059	ApoB	BC075459	BC074467	−1.5	−1.1	−1.6	Restricted (http://Xenopus.nibb.ac.jp/)
Xt_10003855	chordin	CR761722	BC077767	−1.3	−0.6	−1.6	Restricted [50]
Xt_10010647	PDGFRA	CR761598	M80798	−1.3	−0.4	−1.9	Restricted [51]
Xt_10004020	unknown	NM_001015997	BC097726	−1.3	−1.0	−1.3	ND
Xt_10006733	XFz8	DT402720; DT402719	AF017177	−1.3	1.2	−1.5	Restricted [52]

Up regulated genes are shown in green and down regulated genes in red. The Table also shows the RT-PCR data plotted in Fig. 1 and used to validate the microarray results. The data confirm that genes regulated by activin signalling are not regulated by nodal-related signalling, and vice-versa. A description of the expression pattern of each gene is indicated (‘ubiquitous’ or ‘restricted’).

ND: Not determined.

Our experiments identify fewer nodal-regulated genes than the recent microarray study of Sinner and colleagues [26]. This difference probably derives from the facts that Sinner and colleagues harvested embryos at stage 11 rather than 10.5, and defined genes as being differentially expressed if expression levels differed by a factor of 1.4 rather than 2.0. Like Wessely and colleagues, who used a macroarray approach [18], we note that both *Chordin* and *Xsox-17beta* are down regulated by Cerberus-short. We also note that some genes that are down regulated following interference with activin signalling, such as *Xbra* and *goosecoid* [3], were not identified in the present screen. The most likely explanation for this apparent discrepancy is that the expression of such genes is frequently reduced by only 50% or thereabouts [3], and our criteria for defining genes as being differentially expressed (see above) is so stringent that such differences might be regarded as insignificant. RT-PCR analysis of the RNA samples used on the microarrays confirmed previous observations [3] that the expression of these genes is indeed reduced in embryos in which activin signalling is inhibited (data not shown).

### Real-time RT-PCR validation

Our microarray results were validated by real-time RT-PCR The *X. laevis* homologues of the *X. tropicalis* cDNAs recognised by the oligonucleotides (http://informatics.gurdon.cam.ac.uk/cgi-bin/public.exe) were identified by BLAST searches (Table 1), and PCR primers were designed for the great majority of the transcripts that were considered to be differentially expressed. In the case of the activin B experiment, we were unable to identify *X. laevis* homologues for six of the cDNAs, and two primer pairs did not yield a product; in the case of the Cerberus-short experiment, *X. laevis* homologues could not be identified for two cDNAs.

Our RT-PCR analysis used the same RNA samples that were used for microarray experiments. Of the genes tested, 80% of those identified in the activin B experiment were confirmed as being differentially expressed, and all of those identified in the Cerberus-short experiment were similarly verified. Bilateral correlation analysis of the results obtained by microarray hybridization and those obtained by real-time RT-PCR showed a Pearson Correlation of 0.848 (p = 0.000) for the activin B experiment and of 0.975 (p = 0.000) for the Cerberus-short experiment (Fig. 1G,H). RT-PCR experiments confirmed that genes regulated by activin signalling are not regulated by nodal-related signalling, and vice-versa (Table 1). Together, these experiments show that activin and the nodal-related genes regulate distinct genes during early *Xenopus* development.

### Classification of genes regulated by activin and nodal-related genes

**Figure 2:**
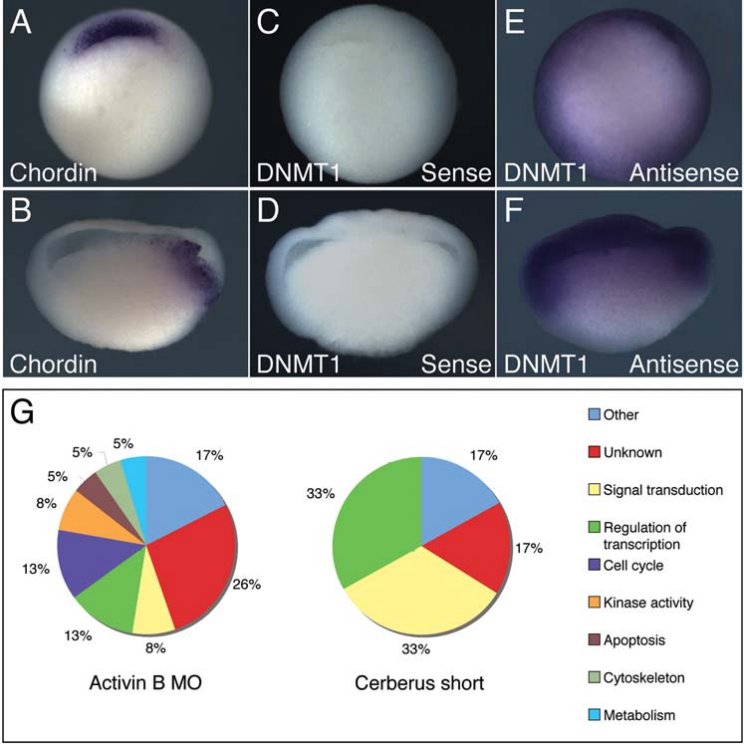
Expression patterns of genes regulated by activin and nodal-related proteins. (A,B) Expression pattern of *Chordin*, a gene that is mis-regulated following inhibition of Xnr signalling. Note that *Chordin* transcripts are restricted to the dorsal marginal zone. (C–F) Expression pattern of DNMT1, a gene that is mis-regulated following inhibition of activin signalling. (C) and (D) show embryos hybridised using a sense probe; (E) and (F) show embryos hybridised using an antisense probe. Note that DNMT1 is expressed ubiquitously.

The expression pattern of each differentially expressed gene was determined from the literature, where possible, or by carrying out in situ hybridisations using *Xenopus tropicalis* embryos with probes generated by the polymerase chain reaction (PCR). Consistent with the different expression patterns of *activin B* and of the *nodal-related* genes [3], [7]–[9], [27], the expression patterns of the genes regulated by the two types of signalling molecules differed (see Table 1). Thus, of the 15 different genes regulated by nodal-related signalling whose expression patterns we know, all are expressed in a restricted fashion (for example, see Fig. 2A,B), and of the 31 genes regulated by activin B, 28 are expressed ubiquitously (for example, see Fig. 2C–F) and three in a restricted fashion.

Genes were then manually classified according to the annotation of their human homologues (NCBI databases, http://www.ncbi.nih.gov/). Interestingly, this analysis also revealed differences between embryos lacking activin B and those in which nodal related signalling is inhibited (Fig. 2G). In particular, while several of the genes regulated by the nodal-related genes are involved in signal transduction or the regulation of transcription, several of the genes whose expression is affected by lack of activin B activity are involved in cell cycle regulation; this is not the case for embryos in which nodal signalling is inhibited.

### Activin regulates cell division in the involuting mesoderm

Both our microarray experiments and our real-time RT-PCR analyses show that down-regulation of activin B, but not loss of nodal-related activity, causes the mis-regulation of genes involved in cell cycle control. One of the effects of the loss of activin B function is a disruption of gastrulation [3], and in this connection we note that the mitotic index of involuting dorsal mesoderm is significantly decreased during gastrulation [28] and that arrest of the cell cycle is required for both bottle cell formation [20] and for convergent extension movements [21], [22]. We therefore asked whether loss of activin B affects cell division during early embryogenesis.

**Figure 3:**
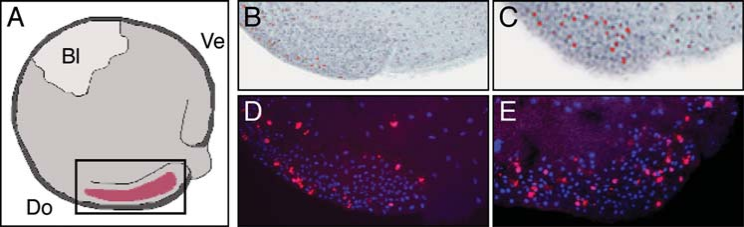
Inhibition of activin B function prevents dorsal axial mesoderm from exiting the cell cycle. (A) Diagram illustrating from which part of the embryo sections in (B–E) are derived. (B,C) Composite images of 10 serial sagittal sections of representative embryos stained with an antibody recognising phosphorylated histone H3 as whole mounts and then sectioned at 12 µm. (B) Control embryo injected with mMO1. Note absence of mitotic cells in involuting mesoderm. (C) Embryo injected with specific antisense oligonucleotide MO3. Involution is perturbed and mitotic cells are visible in dorsal tissue. (D,E) Frozen sections of embryos stained with an antibody recognising phosphorylated histone H3. (D) Control embryo injected with mMO1. Note absence of mitotic cells in involuting mesoderm. (E) Embryo injected with specific antisense oligonucleotide MO3. Involution is perturbed and mitotic cells are visible in dorsal tissue.

Embryos injected with control oligonucleotide mMO1 or specific antisense oligonucleotide MO3 were fixed at the mid gastrula stage and stained using an antibody recognising phosphorylated histone H3, which marks mitotic chromosomes [28]. Inspection of such embryos revealed that the down-regulation of the cell cycle that normally takes place in dorsal axial mesoderm does not occur (Fig. 3). In three control embryos stained as sections the mean mitotic index in dorsal axial mesoderm was 0%; in six embryos injected with MO3 the mitotic index was 12.7±2.7% (mean±standard deviation). Similarly, in a control embryo stained as a whole-mount and then sectioned, the mitotic index was 0%; in an embryo injected with MO3 it was 20%. This failure of the dorsal axial mesoderm to undergo cell cycle arrest is consistent with the observed mis-regulation of cell cycle genes, and it may explain why embryos lacking activin function fail to gastrulate properly [see refs 20]–[22].

## Discussion

Our experiments show that activin B and the nodal-related proteins regulate distinct sets of genes in the early *Xenopus* embryo. In the future it will be interesting to investigate the molecular basis of this difference. One difference between activin and the nodal-related proteins is that their expression patterns differ, with *activin B* being expressed ubiquitously [3], [27] and the nodal-related proteins being restricted to the vegetal and equatorial regions of the embryo [7]–[9]. Consistent with these observations, we note that nodal-regulated genes tend to be expressed in more restricted patterns than do activin-regulated genes (Fig. 2A–F). Another difference is that signalling by the nodal-related proteins, but not activin, requires responding cells to express EGF-CFC family members such as XCR1, 2 and 3 [29]–[32]. This difference between activin and the nodal-related proteins may underlie the ability of activin to activate Smad2 earlier than does Xnr1 or derrière [33]. We note that other studies have also noted differences between activin and nodal signalling; for example, continuous treatment of P19 cells with activin causes only transient activation of Smad2 while treatment with nodal causes sustained activation [32].

Of the genes that are exclusively regulated by activin, several have been implicated in cell cycle regulation (Fig. 2G), and embryos that lack activin B function fail to arrest the cell cycle in dorsal axial mesoderm (Fig. 3). These observations indicate that the role of activin B differs from that of the nodal-related proteins in the early *Xenopus* embryo, and that one of its functions is to control the cell cycle during this critical phase of early *Xenopus* development. This is of importance because axial mesodermal cells arrest the cell cycle after involution [28], and if they are forced to proliferate, this results in a severe disruption of gastrulation [20]–[22]. Interestingly, we note that the ability of activin to inhibit cell division is not restricted to the early *Xenopus* embryos; activin also causes cell growth arrest in human breast cancer cells and in human hepatocytes [34], [35].

We note no effect of the loss of activin on the cell cycle elsewhere in the *Xenopus* embryo; there is no acceleration of cell division in the animal hemisphere, for example, in embryos injected with MO3. It is likely that the cell cycle in the dorsal marginal zone is regulated through locally-acting mRNAs or proteins that require activin signalling for their expression or appropriate post-translation modification.

Finally, what do our results say about the role of activin in mesodermal patterning? Although we emphasise here the role of activin in controlling the expression of genes involved in the regulation of the cell cycle, our previous data, confirmed in the course of the present work (data not shown), indicates that in the absence of activin the expression of genes such as goosecoid, chordin and Xbra is reduced by 20–80%, depending on stage and dose of antisense morpholino oligonucleotide [3]. These observations suggest that activin and the nodal-related proteins (together with Vg1 and derrière) cooperate to specify mesodermal pattern in the embryo, although the results described in this paper argue that the role of activin in this process is less significant than is the role of the Xnrs.

## Materials and Methods

### *Xenopus* embryo manipulations and microinjection

Embryos of *Xenopus laevis* were obtained by artificial fertilisation, maintained in 10% normal amphibian medium [36], and staged as described [37]. For inhibition of nodal-related protein function, embryos were injected at the one cell stage with 600 pg Cerberus-short RNA [17] or, as a control, water. For inhibition of activin B, embryos were injected with 50 ng antisense morpholino oligonucleotide MO3 [3] or, as a control, mMO1 [3]. Embryos were harvested at stage 10.5 for microarray analysis or stage 12 for immunocytochemistry.

### Microarray construction, RNA isolation, labelling and microarray hybridisation

These were performed as described [24].

### Microarray data analysis

Microarray results were imported into Acuity (Axon) and normalised using Lowess normalisation. Data files were created for points which satisfied the following filter: (Sum of Medians) ≥500 AND (Flags) ≥0 AND (%>B532+1SD)≥55 OR (%>B635+1SD)≥55. This filter eliminates data points flagged as bad by GenePix, or that had the sum of media less than 500, or which had fewer than 55% of pixels above background. Points passing these criteria for at least four out of the six microarrays were used for further analysis. Oligonucleotides were considered to be differentially expressed when they showed at least a two fold difference in expression levels in four out of the six microarrays and had a q value of 0 as assessed by the Significance Analysis of Microarrays software [38]. The microarray datasets were deposited in the GEO data repository (http://www.ncbi.nlm.nih.gov/projects/geo/index.cgi) (accession numbers GSE4771 and GSE4777).

### Real time RT-PCR

Differential expression was validated by real-time RT-PCR using the Roche LightCycler 480. Primers specific for *ornithine decarboxylase (ODC)* were as described [3]; others are listed in Table 2.

**Table 2:** RT-PCR primers used in this study.

Qiagen Xt oligo name	Forward primer	Reverse primer
Xt_10000076	CCAGACATTTGTTGCCCTCT	GTTGTGTTGCTGCTGTGCTT
Xt_10000180	TTATGGTGTGGGCAAAGGAC	CTCTTCCCTCTTCATCCTCTTC
Xt_10000182	CCACAGAGTGAAGCACCTGA	AAAACTCAAAAAGAGCCACACTT
Xt_10000293	TGTACCATCGATTTCCAGCA	TCACATGCCAGGCTCTCTG
Xt_10000346	TAAGAAGGCAGTTGCTGCAC	CCTTCTCTAGCCCTTTGTTCA
Xt_10000401	GCGAGAGACAGGTCAAAACC	TTCAATGTCCACCTCCTGGT
Xt_10000572	AGGTGTCCACCTGGTTTGCT	TCAGTGTCTGGGTCATCCAA
Xt_10000615	GCCCCAGAACCACTAAGTAAC	CCTGGACCACCATCTCTGAA
Xt_10000635	AATGGCTTCACGGGTAGATG	AAGCTTTGTCCAGTGCCTTG
Xt_10000757	AGCCCTTCAGATCCACTTCA	GCATCCTCATTTGGATTCGT
Xt_10000971	CCTGAACTGGGAAAAATCCA	AATTCCCATTCCCATGTCAG
Xt_10001337	TCCCTTATATGGGGGTGTGA	GGAACTCATCCTTTGCCTTG
Xt_10002006	GAATGGAGCCCCACAGAATA	TTGCTGATTTGGAACATGGA
Xt_10002067	CTGGACCTGTGGAACTGCTC	CAACAAGCCACGGAAAAACT
Xt_10002154	TCGGATTCCTTATCCAGCAC	GCCTGCATAGCCGTAATCAT
Xt_10002938	GAGATATCCACGGTCAGGTTG	AGCAGAGTAAGGCTGGCAAT
Xt_10003855	AACTGCCAGGACTGGATGGT	GGCAGGATTTAGAGTTGCTTC
Xt_10004020	TCGTCTTGATGGCTGTGTTC	GTGGAGACCTGCATTTCGTT
Xt_10004044	CCTACCCAAGGACAAGGTCA	TGAAAGGCAAACCCACTTTT
Xt_10004134	AGAGTTCCAAACAAACTTGGTG	CTGGCACAGATAGCTGCTCA
Xt_10004273	AAGCCCAAGCTCGTAGAACA	CGGCTGAGCCTTGAATTTAG
Xt_10005146	GATACCGGCATCTCTTCCAA	ATGGTGGAGCGCTTGTTGTA
Xt_10005344	ATGTGGATGTTCCCATCGTT	GTCTGGGCTCATCTCACTGG
Xt_10005362	AACAAGGTCTGCTCCTTCCA	ATGGTGTCTCCACCTCCTTG
Xt_10005487	ACAGATGAGTGTGGGGCAGA	GCTCCACATCAAAGGTCAGG
Xt_10005756	GTGGGCTTCTTCTTCAATGC	GAGTGAGTGCCCAGGATGAT
Xt_10005916	GCTTAAAACTCTCGCCACAGA	TGCTTTAAGCTAAGACCAGGTTG
Xt_10006059	CTTTACATCTGTCCTGCCTCA	TAGTCAGCACCCCTCATCAT
Xt_10006733	GGTGCCCAGCATCAAATCTA	GAACATGCTGCCAATGAACA
Xt_10008086	CACACCAAGTCAAGCAAGGA	TCCTTGCCCACCAACTACAG
Xt_10008633	AGTTCTGCAGGTGGTTTTGG	GCAAGACGGTCATTGAGGTT
Xt_10008637	AAGTTCTGTTATCCCCTGTGC	TTTCTATTGCCACCCAGTCC
Xt_10008667	CGACATGATCCTGTTGGATG	TCTGTGCCCAGATCGATACA
Xt_10008956	CAGAAACTGCTGGTCTGTGC	ATCCCGCTCCTCTATCTTGA
Xt_10008957	GGACTCAATGTGGCTCTTGT	GCCCAACTGTCTCTGAAACC
Xt_10008973	TTTGGAGAGGGATCAGGATG	AGGTATCCTTCCTCAGACAGTTC
Xt_10009006	GTCAAGTCGGAATCCAGCTC	TTCTGCCCCAGGTAGGTACA
Xt_10009228	ATCCGCTCCAATGTTGACTC	GTGAGCAAGGCTTCAATGGT
Xt_10009377	CCTTTCTGACTTTTGCACAGC	GGCAAAGTCTGTTGGATGGT
Xt_10009394	GGTTACAGTTTGCCCACTCC	GTAGGGCATCATCTGGCACT
Xt_10009473	GGCAGAGAACATGGCAAGAG	AGGCCGAATGCATAGATGTC
Xt_10009545	CCAGTCGATGGGCTGTATTT	TTTGTCACCGACAACCTGAA
Xt_10009727	TGGGTCTCCTTCCAGGTGTT	AGGTGGGTGATGGTCCAG
Xt_10009950	CACGGGCTAGAGATTTTCCA	GGCCTCGCTTAGTGTCTTTG
Xt_10010347	AGACAATGCCTGGTGGGTAG	GTTGCCTGGATGGTCTGAAT
Xt_10010647	GAATGGCAAAACCTGACCAT	GCGAGTAACTGCAGGGTGAT
Xt_10010739	CCCCTTATACCCCAAAGAGC	ATGTTGGTCTCCCGTAACAC

### In situ hybridisation

This was carried out on embryos of *Xenopus tropicalis*, essentially as described [39], [40]. Probes were made by use of T7 RNA polymerase; substrates were PCR products obtained using T7 and SP6 primers applied to cDNA clones derived from a large scale *Xenopus tropicalis* EST project [23].

### Immunocytochemistry and Image Acquisition

Embryos to be subjected to frozen sectioning were fixed in 3.7% formaldehyde, 10% DMSO, 100 mM MOPS pH7.4, 2 mM EGTA, 1mM EDTA for 2 hr at room temperature and embedded in 25% sucrose, 15% cold water fish gelatin (Sigma) at room temperature for 24 hr. Sections (14 µm) were cut at −17°C and stored at −80°C. They were incubated overnight at 4°C with anti-phosphohistone H3 antibody (Upstate Biotechnology, 1∶1000) and then with anti rabbit IgG antibody coupled to Alexa 568 (Molecular Probes, A11011, 1∶200). Nuclei were counterstained with DAPI.

Whole-mount immunostaining using anti-phosphohistone H3 antibody was performed as described [28].
